# The transition to fully open access: a new era for the Journal of Clinical Monitoring and Computing

**DOI:** 10.1007/s10877-025-01342-7

**Published:** 2025-08-12

**Authors:** Moritz Flick, Francisco A. Lobo

**Affiliations:** 1 Department of Anesthesiology, Center of Anesthesiology and Intensive Care Medicine, University Medical Center Hamburg-Eppendorf, Martinistrasse 52, 20246 Hamburg, Germany; 2 Anesthesiology Department, Yas Clinic Khalifa City– Abu Dhabi Stem Cells Center, Abu Dhabi, UAE

The *Journal of Clinical Monitoring and Computing* will become a fully open access Journal in 2026. Since its founding in 1985, the Journal has served as a vital platform for researchers, clinicians, engineers, and other professionals advancing perioperative and critical care monitoring. The *Journal of Clinical Monitoring and Computing* is a well-established journal in Anesthesiology and Intensive Care Medicine that brings together experts from diverse backgrounds and fields– always keeping the patient at the center of attention. Our mission remains the same: to provide a rigorous platform for innovations that ultimately improve patient outcomes.

The traditional subscription model of scientific journals historically served to fund production and distribution. It also made scientific publishing a profitable business model, with high profit margins that are unmatched by other industries [1]. Today, the subscription model has rather become a barrier that contradicts the very purpose of scientific publishing, as subscription-based models have created significant obstacles to knowledge dissemination. When clinicians cannot access the latest research due to institutional paywalls, evidence-based medicine suffers. Financial barriers restrict access to valuable clinical insights, creating disparities that hinder scientific discourse and sustain inequalities in global healthcare knowledge access.

**Fig. 1:**
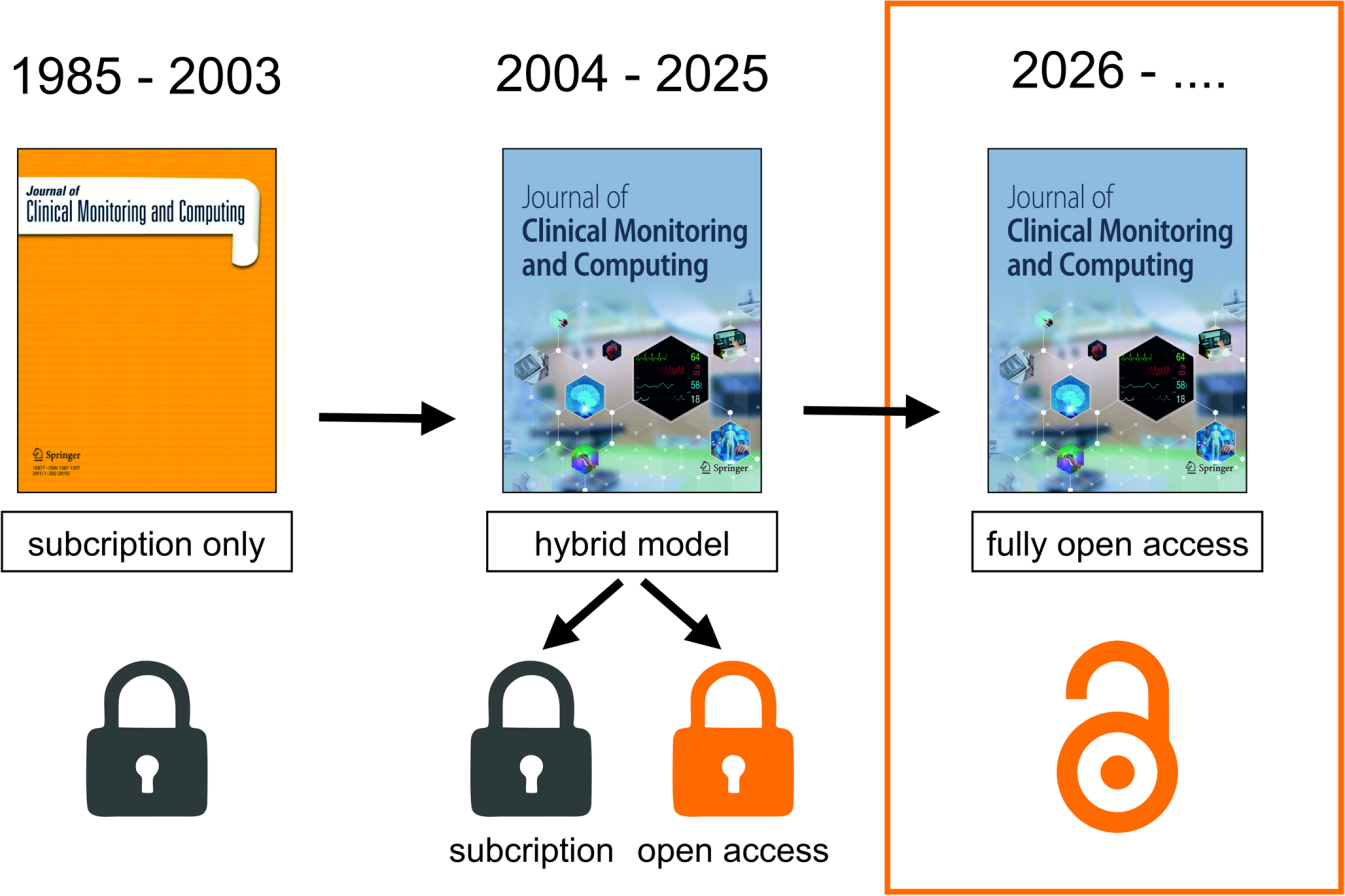
Transition of the Journal of Clinical Monitoring and Computing from subscription to open access

In response to pressure from the academic community and funding agencies, many journals– including the *Journal of Clinical Monitoring and Computing*– adopted hybrid models that offer both subscription-based access and open access choices. In this system, authors can choose to pay a publishing fee to make their article open access or publish without cost, with their work behind a paywall. Since hybrid publishing was introduced, the percentage of open access articles in the *Journal of Clinical Monitoring and Computing* has risen to 45% in 2024– with 66% of these funded through transformative agreements like Project DEAL [2]. Although this hybrid approach was well-intentioned, it created a paradoxical situation where institutions pay both subscription fees and publication charges. We, therefore, welcome Springer Nature’s decision to make the *Journal of Clinical Monitoring and Computing* a fully open-access journal starting in 2026 (Fig. 1). Fully open access publishing boosts research visibility, promotes worldwide knowledge sharing, and gives unrestricted access to scientific literature for healthcare professionals and the general public [3].

The clinical implications of this transition go well beyond publishing logistics. Improved global knowledge sharing allows for quick adoption of evidence-based practices across different healthcare settings. Greater visibility of clinical innovations speeds up their use in patient care, while open access enables healthcare providers worldwide to utilize the latest monitoring technologies and techniques. Several funding organizations and governments now require that publicly funded research be made immediately available without delay, recognizing the direct link between research accessibility and public health outcomes.

Springer Nature’s decision to move the *Journal of Clinical Monitoring and Computing* to fully open access reflects these changing expectations and the growing need for unrestricted scientific communication. Although the editorial team had no role in this publisher-driven decision, we recognize both the opportunities and challenges it brings. Our main concern as clinicians remains scientific rigor and patient safety, and we acknowledge valid concerns about some fully open access publishers that promote rapid publication processes potentially at the expense of scientific quality. The implementation strategy addresses potential barriers through several mechanisms designed to support global healthcare equity. Article publishing charges will be waived or reduced for authors from lower-middle income countries, ensuring that geographical location does not determine research participation or access to clinical innovations. Editorial vouchers will allow for a limited number of articles to be published without charge, providing additional support for valuable research that might otherwise face financial constraints [4].

While initially challenging, the integration of Springer Nature’s SNAPP peer review system has already demonstrated its value in streamlining our editorial process without compromising quality. Similarly, we anticipate that the open access transition, properly managed, will ultimately strengthen rather than weaken our journal’s impact.

The growing interest in *Journal of Clinical Monitoring and Computing* publications over recent years confirms our editorial approach. Moving to fully open access should speed up this trend, making our content more available to perioperative and critical care professionals worldwide. We also invite our valued readers and contributors to connect with us via social media, where we regularly share interviews and highlights of recent publications that support our collective dedication to evidence-based patient care.

We remain committed to providing a rigorous, accessible platform for research that directly impacts clinical practice and patient safety. Our mission continues unchanged: facilitating the exchange of clinically relevant innovations in monitoring technology that enhance patient care quality and safety.
